# Translation and Cultural Adaptation of the Pictorial Fit-Frail Scale for Health Care Professionals Into Canadian-French

**DOI:** 10.1177/30495334251391393

**Published:** 2025-10-31

**Authors:** Jade Gagnon, Danica Brousseau, André Bussières, Julie-Marthe Grenier, Isabelle Pagé

**Affiliations:** 1 Université du Québec à Trois-Rivières, QC, Canada; 2 McGill University, Montréal, QC, Canada; 3 Centre Interdisciplinaire de Recherche en Réadaptation et Intégration sociale (Cirris), Québec City, QC, Canada

**Keywords:** frailty, geriatric assessment, translations, cultural adaptation, health occupations

## Abstract

Frailty is a clinical syndrome linked to increased vulnerability in older adults. The Pictorial Fit-Frail Scale for Health Care Professionals (PFFS-HCP) is a visual tool assessing frailty across multiple domains, but no French version is currently available. This study aimed to translate and culturally adapt the PFFS-HCP into Canadian French for use in Canada. Following adapted cross-cultural guidelines, three bilingual translators independently translated the 33-item data dictionary. Discrepancies were resolved by an expert panel through consensus, and the final version was submitted to the original developers and formatted by their design team. Of the items, 36.4% were identical across translations, 33.3% matched in two versions, and 30.3% differed in all three. Two items were adapted from the French Clinical Frailty Scale. The final Canadian French version of the PFFS-HCP is now available. A follow-up study will assess its clarity and usability in Québec clinical settings.

## Introduction

Frailty is a multidimensional clinical syndrome characterized by diminished physiological reserves and increased vulnerability to stressors, particularly among older adults (Cohen et al., 2023; Oliveira et al., 2020). This syndrome is associated with adverse outcomes such as functional decline, falls, prolonged hospitalizations, and increased mortality (Oliveira et al., 2020). Early identification of frailty has been reported to be crucial for guiding clinical decision-making, tailoring care plans, and improving patient outcomes (Cohen et al., 2023).

Assessing frailty can be challenging for healthcare professionals due to time constraints, limited training, and the complexity of existing assessment tools. The Pictorial Fit-Frail Scale (PFFS) was developed to address these challenges (Theou et al., 2019). It is a visual tool that evaluates frailty across 14 domains of health and function using pictograms instead of text-based questions (Theou et al., 2019). This design makes it accessible across literacy levels, languages, and cultures and allows for rapid administration, typically under 2 min for health care professionals and 5 min for patients (McGarrigle et al., 2019; Theou et al., 2019). A version of the PFFS has been adapted for completion by healthcare professionals to assess either the patient’s usual state of frailty (PFFS-HCP) or their acute/current state (PFFS-HCP Acute), while other versions have been designed for self- or proxy-assessment by patients or caregivers (Dalhousie University, n.d.). The PFFS has demonstrated validity and reliability across various contexts including clinical settings (Ahip et al., 2021), and geriatric outpatient clinics (McGarrigle et al., 2019). Depending on the version of the PFFS, the tool is available in more than six languages, facilitating its global dissemination and use in diverse clinical and cultural contexts (Dalhousie University, n.d.).

Despite its growing international adoption, no French version of the PFFS-HCP was available to date. This represents a significant gap, particularly in Canada, where French is one of the two official languages. Our research team undertook the translation and cultural adaptation of the PFFS-HCP into Canadian French, following established methodological guidelines. This report outlines the steps taken to ensure linguistic accuracy, cultural relevance, and fidelity to the original instrument’s intent.

## Methods

**Figure 1.**
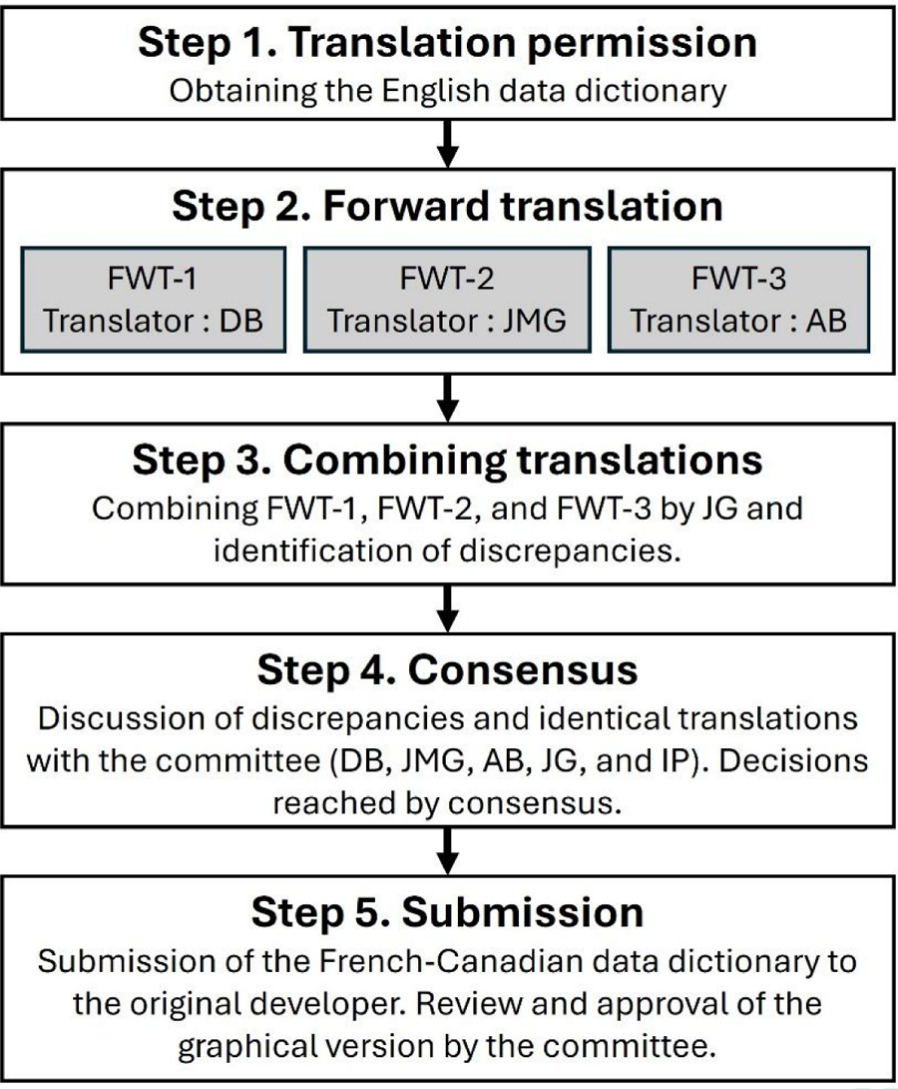
Flow diagram illustrating the cross-cultural adaptation process.

A flow diagram illustrating the cross-cultural adaptation process is provided in Figure 1. This study followed an adapted version of the guidelines proposed by Sousa and Rojjanasrirat (Sousa & Rojjanasrirat, 2011). The process included the following steps: (1) obtaining permission for translation and access to the data dictionary, (2) conducting the forward translation, (3) combining translations and identifying discrepancies, (4) reaching consensus on discrepancies between translations, (5) sharing the translated data dictionary with the original developers. As suggested by McKown et al. (McKown et al., 2020), no pilot testing or cognitive interviewing involving patients or healthcare professionals was conducted, given that the PFFS-HCP is a clinician-reported outcome measure. Notably, the back-translation step was omitted, in accordance with the recommendations of Epstein et al. (Epstein et al., 2015), who argue that back-translation may not always be necessary when the translation process involves expert consensus and contextual adaptation. The IRICA (Improving the Reporting Quality of Instrument Cross-Cultural Adaptation) statement was used as a framework to guide the reporting (Hou et al., 2021).

### Step 1. Translation Permission and Data Dictionary

The PFFS-HCP was developed by the Geriatric Medicine Research (GMR) team at the Department of Medicine, Dalhousie University (Halifax, Nova Scotia, Canada). Permission to translate the instrument into Canadian French was requested through their online Permission Request Portal and was granted on May 2, 2025 (Request ID: 250319530). The GMR team provided a data dictionary in .DOC format, consisting of 33 entries. As the instrument is primarily pictorial rather than text-based, the elements requiring translation were mostly short phrases, with the exception of instrument title, general instructions, and statement regarding translation permissions.

### Step 2. Forward Translation

Although standard guidelines recommend involving two bilingual individuals, one familiar with the instrument and one unfamiliar, for independent translation (Sousa & Rojjanasrirat, 2011), our research team opted to include three bilingual individuals with diverse professional backgrounds to enrich the translation process. All three translators were native Canadian French speakers and were professors in the Department of Chiropractic at Université du Québec à Trois-Rivières (UQTR). JMG holds a PhD with expertise in qualitative research and radiology but has no specific background in frailty. DB holds a certificate in gerontology, teaches geriatric chiropractic care, and maintains a limited clinical practice mostly caring for aging patients. AB is a senior researcher in knowledge translation and had no prior familiarity with the instrument.

Each translator received the data dictionary in .DOC format, along with the English version of the instrument, which is publicly available (Dalhousie University, n.d.). They were instructed to translate each item into Canadian French, bearing in mind that the instrument is intended for use by a wide range of healthcare professionals, including but not limited to chiropractors. Additionally, they were encouraged to suggest any cross-cultural adaptations they deemed necessary to ensure the translated version would be both linguistically accurate and culturally appropriate. At the end of this step, three translated versions were sent back to the research team (FWT-1, FWT-2, and FWT-3).

### Step 3. Combining Translations and Identifying Discrepancies

One member of the research team (JG) compiled the three translated versions into a single Excel (.XLS) file, anonymizing the contributions so that the identity of each translator remained undisclosed. At the time of the study, JG was pursuing a master’s degree, while practicing as a chiropractor in private practice. All comments provided by the translators were also consolidated into this document. JG then categorized the entries into three groups: those with identical translations across all three versions, those with identical translations from two out of three translators, and those with three distinct versions.

### Step 4. Reaching Consensus

A 1-hr online meeting was held with the three translators, the principal investigator (IP), and the researcher responsible for compiling and analyzing the translations (JG). IP is a professor in the Department of Chiropractic at UQTR, with research expertise in the biomechanics and safety of manual therapy in the aging population. During this meeting, all entries were reviewed, including those for which consensus had already been reached. Translator comments regarding potential cross-cultural adaptations were also discussed. For entries with discrepancies, final decisions were made through a consensus-based approach.

### Step 5. Sharing With Original Developers

The finalized translated data dictionary was submitted to the original developers who forwarded the content to their graphic design team. Proofs of the adapted instrument were then sent back to the research team for review. After final approval, the GMR team made the Canadian-French version of the PFFS-HCP publicly available.

## Results

### Step 3. Combining Translations and Identifying Discrepancies

Of the 33 entries in the data dictionary, 12 (36.4%) were identical across all 3 translations, 11 (33.3%) were identical in 2 of the 3 versions, and the remaining 10 entries (30.3%) had 3 distinct translations. One specific comment related to cross-cultural adaptation was reported by the translators.

### Step 4. Reaching Consensus

All discrepancies were resolved through consensus during a team meeting. Two of the entries with discrepancies were finalized using wording from another instrument on the GMR website, the Clinical Frailty Scale (CFS) already available in Canadian French. These entries concerned the permission for translation and the identification of the research team responsible for the adaptation. The name of the instrument was also determined by consensus. It combined elements from the three proposed versions and resulted in the following title: *Échelle Illustrée de Condition Physique et de Fragilité (EICPF), version professionnel de la santé (PF)*. Interestingly, a few discrepancies were found to be related to the use of anglicisms or words borrowed from English. These words may or may not keep the same meaning when brought into French. For example, the team discussed the difference between the words “score” (both used in French and English and “results” (“*résultats*” in French). Another example of an element that was adapted would be the item “Vision (with glasses if needed).” This item was revised to enhance inclusivity, resulting in the translation “*Vision (corrigée au besoin)*,” which translates to “Vision (corrected if needed).” The final bilingual data dictionary, including the original English entries and their Canadian-French translations, is presented in Table 1.

**Table 1.** Final Data Dictionary Including the Original Source Language and the Translated Version of the 33 Entries.

Original source language	Translation in French Canadien
PFFS VERSION 1.0 Health Care Professional - English	EICPF VERSION 1.0 Professionnel de la Santé – Français
Pictorial Fit-Frail Scale	Échelle Illustrée De Condition Physique et de Fragilité
PFFS	EICPF
Health Care Professional	Professionnel de la santé
HCP Version	Version PS
Name:	Nom:
Date:	Date:
Total Score:	Résultat total:
Score	Résultat
For each category, choose ONE picture that is closest to your patient’s USUAL state. Circle the score below that picture and transfer it to the right.	Pour chacune des catégories, choisir UNE image qui représente le plus fidèlement l’état HABITUEL de votre patient. Encercler le résultat situé sous l’image et le reporter dans la colonne de droite.
Best	Meilleur
Worst	Pire
1 MOOD	1 HUMEUR
2 NUMBER OF MEDICATIONS	2 NOMBRE DE MÉDICAMENTS
3 MOBILITY	3 MOBILITÉ
4 FUNCTION	4 CAPACITÉ FONCTIONNELLE
5 BALANCE	5 ÉQUILIBRE
6 SOCIAL CONNECTIONS	6 RELATIONS SOCIALES
7 DAYTIME DROWSINESS	7 SOMNOLENCE DIURNE
8 MEMORY AND THINKING	8 MÉMOIRE ET FONCTIONS COGNITIVES
9 VISION (WITH GLASSES IF NEEDED)	9 VISION (CORRIGÉE AU BESOIN)
10 HEARING (WITH HEARING AID IF NEEDED)	10 AUDITION (AVEC APPAREIL AUDITIF AU BESOIN)
11 PAIN	11 DOULEUR
12 UNINTENTIONAL WEIGHT LOSS	12 PERTE DE POIDS NON INTENTIONNELLE
13 AGGRESSIONS	13 AGRESSIVITÉ
14 BLADDER CONTROL	14 CONTRÔLE URINAIRE
Page 1 of 2	Page 1 de 2
Page 2 of 2	Page 2 de 2
Page 1 Score	Résultat de la page 1
Page 2 Score	Résultat de la page 2
For permission: www.geriatricmedicineresearch.ca	Pour permission: www.geriatricmedicineresearch.ca
© Theou and Rockwood PFFS HCP Version 1.0	© Theou and Rockwood PFFS HCP Version 1.0
Translated with permission into [language] by [name], [place], [year]	Traduit avec permission vers le français par Isabelle Pagé et collaborateurs, Trois-Rivières (Québec), Canada, 2025

### Step 5. Sharing With Original Developers

The translation and cultural adaptation process led to the development of a finalized French-Canadian version of the PFFS-HCP. Following final approval of the graphical version by the research team, the adapted instrument was officially published and made publicly available in July 2025 through the GMR team’s online Permission Request Portal: https://www.dal.ca/sites/gmr/our-tools/permission-for-use.html.

## Discussion

The translation and cultural adaptation of clinical assessment tools are increasingly recognized as critical steps in ensuring their effectiveness, accuracy, and equity across diverse healthcare settings (Epstein et al., 2015; McKown et al., 2020). As healthcare becomes more globalized and patient populations more multicultural, the need for tools that are both linguistically and culturally appropriate has grown substantially. Tools developed in one language or cultural context may not be directly applicable in another without risking misinterpretation, reduced validity, or even harm to patients (Epstein et al., 2015; McKown et al., 2020). Our team is based in Quebec and consists of French Canadians, where 84.1% of Canada’s French-speaking population lives (The Department of Canadian Heritage, 2024). Although terminology may vary slightly in other provinces, this adaptation can be used by healthcare practitioners in other provinces. Properly adapted tools help ensure that assessments are understood as intended, preserve the psychometric properties of the original instrument, and support consistent data collection across populations (Epstein et al., 2015; McKown et al., 2020).

The GMR team at Dalhousie University has supported the translation of several of its tools, including the CFS and the PFFS, into multiple languages (Dalhousie University, n.d.). These efforts reflect a broader commitment to making frailty assessment tools accessible and usable in diverse clinical environments. Our approach to translating the PFFS-HCP into Canadian-French aligns with this methodology. Although the GMR team does not provide specific guidance on translation procedures, the process typically involves local experts, bilingual translators, and consensus-based decision-making.

In our case, the high level of agreement among translators and the use of reference phrasing from the Canadian-French version of the CFS (Dalhousie University, n.d.) ensured both linguistic accuracy and cultural appropriateness. Unlike some adaptations that include full psychometric validation, our process focused on linguistic and contextual fidelity. However, as with other GMR tools, the next step involves field testing. A study involving chiropractors in Canada will assess for clarity and usability in clinical practice, contributing to the growing body of evidence on the practical implementation of frailty assessment tools in diverse healthcare environments.

### Limitation

The main limitation of this study is that the procedure was limited to the translation and cultural adaptation of the PFFS-HCP into Canadian-French. While linguistic accuracy and cultural appropriateness were ensured, full psychometric validation, including reliability and construct validity, has not yet been conducted. Future studies will be needed to assess these properties and confirm the scale’s use in clinical practice.

## Conclusion

The Canadian-French version of the PFFS-HCP was successfully translated and culturally adapted through a rigorous and collaborative process. The final version is now publicly available and ready for use by healthcare professionals in Canada. While formal psychometric validation was not conducted during the translation phase, a follow-up study will assess the tool’s clarity and usability in clinical practice. These efforts contribute to the broader goal of improving frailty assessment and care for aging adults through accessible and culturally appropriate tools.
